# Spectrum of hemoglobinopathies with hematological and biochemical profile: A five year experience from a tertiary care hospital

**DOI:** 10.12669/pjms.38.8.5935

**Published:** 2022

**Authors:** Neelum Mansoor, Fatima Meraj, Ameerah Shaikh, Naeem Jabbar

**Affiliations:** 1 Neelum Mansoor, FCPS. Consultant, Department of Hematology and Blood Center, Indus Hospital and Health Network, Karachi, Pakistan; 2 Fatima Meraj, FCPS. Section Head, Department of Hematology and Blood Center, Indus Hospital and Health Network, Karachi, Pakistan; 3 Ameerah Shaikh, Medical Student, Ziauddin University Hospital, Karachi, Pakistan; 4 Naeem Jabbar, FCPS. Consultant, Blood Centre, Indus Hospital and Health Network, Karachi, Pakistan

**Keywords:** Beta thalassemia, Hemoglobinopathies, High performance liquid chromatography (HPLC), Hb E disease

## Abstract

**Background & Objective:**

Determination of hemoglobinopathies is significant for epidemiological studies. There is a need to identify burden of hemoglobinopathies at national level to lay down the foundation of appropriate screening and prevention programs. The present study aimed to evaluate the spectrum of hemoglobinopathies along with hematological and biochemical parameters in a tertiary care hospital.

**Methods:**

This retrospective study included results of high performance liquid chromatography (HPLC) test from July 2015 - May 2020 in the department of Hematology, Indus Hospital and Health Network, Karachi, Pakistan. Data of all patients collected for red blood cell (RBC) indices, serum iron profile, and vitamin B12 and red cell folate levels. Diagnosis of hemoglobinopathies was done by an automatic analyzer ADAMS A1C Model No. HA-8180T Arkray/Japan.

**Results:**

Among 2422 participants, hemoglobinopathy observed in 14.5% (n=352). Beta thalassemia trait is observed as the most common hemoglobinopathy (6.4%). Severe anemia (Hb=5.1-5.5 g/dl) found in beta thalassemia major (BTM) and HbE disease. Red cell parameters showed significant association with different types of hemoglobinopathies. Mean ferritin level was high in E-beta thalassemia (687.8±591.9) followed by sickle cell disease (615.7±543.5).

**Conclusion:**

Apparently, overall frequency is static however, results of this study are not applicable to general population due to sample bias. Moreover, true figures are difficult to identify due to high incidence of iron deficiency anemia that masks the diagnosis by conventional techniques. Molecular characterization by DNA analysis is the most reliable tool of diagnosis. However, this method is not widely available in our country due to lack of expertise and cost issues.

## INTRODUCTION

Hemoglobinopathies are the most frequent genetically inherited disorders.^1^ β-TM and sickle cell disease (SCD), have been studied in depth due to high mortality and morbidity associated with these two disorders.^2^ Globally, around 240 million cases of beta thalassemia trait (β-TT) are diagnosed annually, in Southeast Asian countries and Mediterranean region. The distribution of hemoglobinopathies varies geographically and by community. Of all the hemoglobin disorders, beta thalassemia (β-Thal) is the most common single gene disorder in Pakistan with an estimated pool of 9.8 million carriers in local habitants and ~50,000 of the patients receiving treatment in various centers countrywide. There is about 5-8% prevalence of β-Thalassemia trait in Pakistan which results in an estimated rate of 5000-9000 births with β-Thalassemia major per year. These numbers can be a real challenge for the health care system of a low and middle income country like Pakistan.^3^,^4^

More than 700 hemoglobin variants involving genes both from alpha and beta gene clusters have been identified. Among these, the thalassemia comprise a heterogeneous set of disorders, depending on insufficiently synthesized globin chain(s).^5^ In addition to the transfusion-dependent form of β-Thal, there are milder forms that may go undetected until adulthood. Because of their high frequency and severity, β-TM pose the most serious public health concern. HbE disease and E trait are monogenic diseases that are distributed throughout the Mediterranean, the Middle East, and the Indian subcontinent.

Several countries have implemented preventive screening programs worldwide to lower the prevalence of hemoglobinopathies.^6^ A premarital screening program was established in 1976 in Rome to detect β-TT.^7^ In 1980, SCD preventive program was established in Virginia.^8^ In Pakistan there is no such effort made at national level while some of non-governmental organizations are actively working under thalassemia federation of Pakistan. There is a need to start addressing the burden of this disease on health care system at national level to lay down the foundation of appropriate screening program. The present study was aimed to see the pattern of hemoglobinopathies in a tertiary care hospital. Red cell parameters and biochemical profile not only play a significant role in diagnosis of hemoglobinopathies but also impact the disease course of these patients therefore it is also incorporated in analysis.

## METHODS

This retrospective cross sectional study conducted in the hematology department of the Indus Hospital and Health Network, Karachi, Pakistan. The study was approved from institutional review board (IRB) of Indus Hospital Research Centre (IRD_IRB_2017_12_008). Data of all cases requested for hemoglobinopathy testing was reviewed in electronic medical record from July, 2015 to May 2020. Among the retrieved 2436 cases of all age groups and sex, 2422 were included in this study. Remaining 14 cases were excluded due to the absence of hemogram. Repetition i.e., samples received more than once for HPLC were carefully excluded. Diagnosis of hemoglobinopathies was done by High Performance Liquid Chromatography (HPLC) an automatic analyzer ADAMS A1C Model No. HA-8180T Arkray/Japan. Blood sample (3ml) was taken in ethylenediaminetetraacetic acid (EDTA) anti-coagulated evacuated tube for complete blood count (CBC) and HPLC. Testing for CBC to obtain red blood cell (RBC) parameters was done by automated hematology analyzer, Sysmex XN1000. Hemoglobin (Hb), RBC count, hematocrit (HCT), mean corpuscular volume (MCV) and mean corpuscular hemoglobin (MCH), mean corpuscular hemoglobin concentration (MCHC) were recorded. Biochemical parameters including ferritin, serum iron, total iron binding capacity (TIBC), transferrin, serum vitamin B12 and red cell folate levels also documented. Serum ferritin including iron profile was done on Abbott Alinity-C, serum vitamin B12 tested on Abbott Alinity-I and red cell folate performed on Roche Cobas E411. A parallel sickling test using the reducing agent sodium metabisulfite was done for cases with an abnormal hemoglobin in the S window of the chromatogram. Data was analyzed using SPSS 23. Mean ± SD was calculated for age and red cell parameters. Association of diagnosis with hemogram was calculated using one way ANOVA test.

## RESULTS

**Fig.1:**
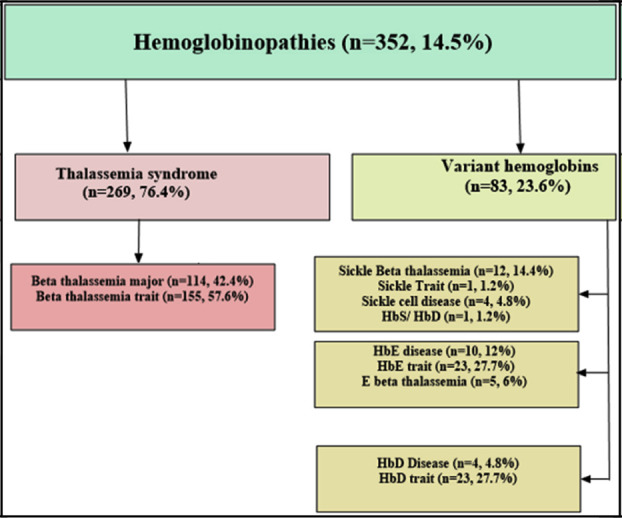
Frequency of hemoglobinopathies.

Among 2,422 participants, hemoglobinopathy was observed in 14.5% (n=352) cases. Mean age of patients with hemoglobinopathy was 11.7±13.5 years with 0.5:1 M/F ratio. Study participants were grouped according to age, of which >15-40 years group constituted the predominant one (52%). Amongst 352 cases of hemoglobinopathy, 76.4% (269/352) were of β-Thal while the remaining harbored variant hemoglobin 23.6% (n=83/352) (Fig.1). Out of 2422 cases, thalassemia syndrome showed the highest frequency (11.1%) with β-TT constituted the major proportion (6.4%) while compound heterozygosity was the lowest in frequency (0.04%).

Mean ferritin level was high in E-beta thalassemia (687.8±591.9) and SCD (615.7±543.5) followed by sickle beta thalassemia (562.9±698.1). In patients with no hemoglobinopathy, decreased ferritin observed in 55.4% cases however 76.1% showed decreased levels of iron, 20.9% revealed reduced serum vitamin B12 while 15% had low red cell folate levels (Table-I).

**Table-I:** Biochemical profile of study participants according to diagnosis.

Diagnosis	Categories	Ferritin (20-200µg/ml)	Iron (59-158 µg/dL)	TIBC (295-388µg/dL)	Transferrin saturation (20%-40%)	B12 (174-878pg/ml)	Folate (Pediatric >160ng/ml, Adult 140-628ng/ml )
No hemoglobinopathy	Decreased	1002/1810 (55.4)	726/954 (76.1)	218/701 (31.1)	525/695 (75.5)	100/478 (20.9)	46/303 (15)
	Normal	631/1810 (34.9)	165/954 (17.3)	172/701 (24.5)	90/695 (12.9)	333/478 (69.7)	227/303 (75)
	Raised	177/1810 (9.8)	63/954 (6.6)	311/701 (44.4)	80/695 (11.5)	45/478 (9.4)	30/303 (10)
Beta Thalassemia major	Decreased	22/93 (23.7)	14/77 (18.2)	32/47 (68.1)	7/46 (15.2)	2/24 (8.3)	2/12 (17)
	Normal	28/93 (30.1)	27/77 (35.1)	11/47 (23.4)	9/46 (19.6)	20/24 (83.3)	10/12 (83)
	Raised	43/93 (46.2)	36/77 (46.8)	4/47 (8.5)	30/46 (65.2)	2/24 (8.3)	-
Beta Thalassemia trait	Decreased	48/135 (35.6)	38/86 (44.2)	33/63 (52.4)	32/63 (50.8)	10/38 (26.3)	3/27 (11)
	Normal	48/135 (35.6)	36/86 (41.9)	16/63 (25.4)	11/63 (17.5)	19/38 (50.0)	19/27 (70.4)
	Raised	39/135 (28.9)	12/86 (14.0)	14/63 (22.2)	20/63 (31.7)	9/38 (23.7)	5/27 (18.5)
HbD trait	Decreased	11/20 (55.0)	9/13 (69.2)	2/12 (16.7)	9/12 (75.0)	-	1/4 (25)
	Normal	7/20 (35.0)	3/13 (23.1)	4/12 (33.3)	2/12 (16.7)	5 (100)	3/4(75)
	Raised	2/20 (10.0)	1/13 (14.0)	6/12 (50.0)	1/12 (8.3)	-	
Hb D disease	Decreased	-	-	1/2 (50.0)	-	-	-
	Normal	2/4 (50.0)	2/2 (100)	1/2 (50.0)	1/2 (50.0)	1 (100)	-
	Raised	2/4 (50.0)	-	-	1/2 (50.0)	-	-
Sickle-beta Thalassemia	Decreased	-	1/7 (14.3)	3/4 (75.0)	2/4 (50.0)	-	-
	Normal	5/8 (62.5)	6/7 (85.7)	1/4 (25.0)	2/4 (50.0)	1 (100)	-
	Raised	3/8 (37.5)	-	-	-	-	-
Sickle cell Disease	Decreased	-	-	1/1 (100)	-	-	-
	Normal	-	-	-	-	1 (100)	1/1 (100)
	Raised	2/2 (100)	-	-	1/1 (100)	-	
HbS & HbD Disease	Decreased	1/1 (100)	-	-		-	-
	Normal	-	-	-		1 (100)	-
	Raised	-	-	-		-	-
HbE trait	Decreased	6/17 (35.3)	5/7 (71.4)	3/5 (60.0)	4/5 (80.0)	-	1/2 (50)
	Normal	8/17 (47.1)	2/7 (28.6)	2/5 (40.0)	-	3 (100)	1/2 (50)
	Raised	4/17 (17.6)	-	-	1/5 (20.0)	-	
E-Beta Disease	Decreased	-	2/3 (66.7)	2/3 (66.7)	-	-	1/1 (100)
	Normal	1/4 (25)	1/3 (33.3)	1/3 (33.3)	3/3 (100)	-	-
	Raised	3/4 (75)	-	-	-	-	-
Hb E disease	Decreased	3/9 (33.3)	3/8 (37.5)	3/6 (50.0)	1/6 (16.7)	1/3 (33.3)	-
	Normal	1/9 (11.1)	2/8 (25.0)	2/6 (33.3)	2/6 (33.3)	2/3 (66.7)	-
	Raised	5/9 (55.6)	3/8 (37.5)	1/6 (16.7)	3/6 (50.0)	-	5/5 (100)

**Fig.2:**
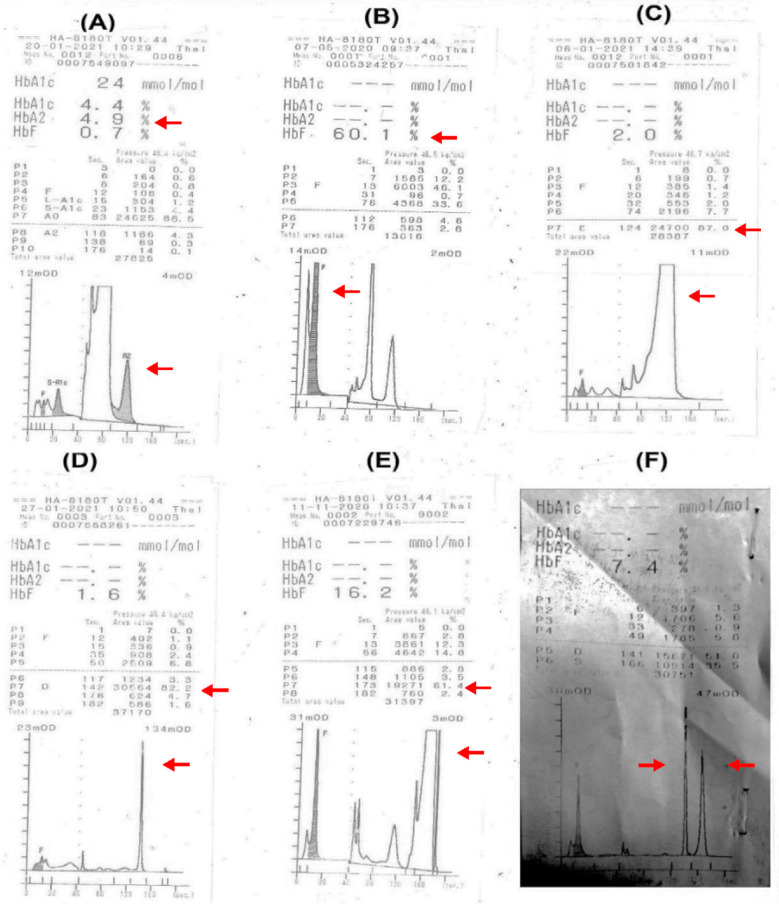
Typical elution patterns (chromatograms) for various hemoglobinopathies with Arkray HA-8180 high performance liquid chromatography (A) Beta thalassemia trait (B) Beta thalassemia major (C) Hb E disease (D) Hb D disease (E) Sickle cell disease (F) Compound heterozygosity for HbS/HbD.

Severe anemia observed in β-TM (Hb=5.5±2.1) followed by HbE disease (Hb=5.8±3.0). Among all hemoglobinopathies, β-TT showed the highest red blood cell count (5.2±10.9). The lowest mean values of MCV and MCH were observed in HbD disease, 58.6 and 18.4 respectively. Β-TM cases exhibited the highest red cell distribution width (RDW=36.6±41.5). There can be a possible contributing effect of concurrent nutritional deficiency on RDW. As shown in Table-II, nutritional deficiency workup was not performed in all the cases of B-TM. However, among the available data, 24%, 8% and 17% cases of B-TM showed concurrent deficiency for ferritin, B12 and folate levels respectively. Significant association was observed between Hb, HCT, MCV, MCH, MCHC and diagnosis (Table-II). Typical elution patterns (chromatogram) of significant hemoglobinopathies are depicted in Fig.2.

**Table-II:** Association between diagnosis & hemogram (Mean ± SD).

Diagnosis	RBC	p-value	Hb	p-value	HCT	p-value	MCV	p-value	MCH	p-value	MCHC	p-value	RDW	p-value
No hemoglobinopathy	4.5±6.6	0.27	8.5±2.7	<0.001	28.4±7.8	<0.001	70.0±13.7	0.004	21.3±5.6	<0.001	30.0±4.0	<0.001	22.6±43.9	0.51
Beta thalassemia trait	5.2±10.9		8.0±2.7		25.6±8.9		72.6±17.6		23.1±6.4		32.1±6.2		21.5±9.7	
Beta thalassemia major	2.5±1.0		5.5±2.1		17.3±6.8		71.2±14.2		23.1±5.3		32.6±4.7		36.6±41.5	
HbD trait	4.19±0.9		9.3±2.4		29.2±6.9		69.7±8.5		22.3±3.7		31.7±2.0		32.4±52.6	
HbD disease	3.9±1.0		7.2±1.9		22.9±5.2		58.6±5.0		18.4±1.2		31.4±1.5		34.1	
HbE trait	4.1±1.2		8.5±2.8		26.9±7.7		65.9±9.6		20.7±4.1		31.2±3.1		32.6±45.6	
E-beta thalassemia	3.3±0.7		8.0±2.2		24.7±5.8		74.4±10.2		23.8±3.8		32.1±2.5		22.5±3.3	
HbE disease	2.8±1.2		5.8±3.0		18.6±8.5		67.4±16.6		21.2±6.5		31.1±2.7		28.9±4.8	
HbS trait	2.9		7.6		24.1		83.1		26.2		31.5		-	
Sickle cell disease	2.5±0.5		7.8±1.8		23.3±4.4		92.3±10.3		30.8±4.7		33.3±1.7		14.7±10.3	
Sickle-beta thalassemia	3.7±0.3		6.4±0.9		22.2±2.8		60.2±10.6		17.4±3.2		29.0±1.0		31.9	
HbS & HbD	1.8		5.7		18.1		96.8		30.5		31.5		23.9	

## DISCUSSION

The overall frequency of hemoglobinopathies in this study was 14.5% (n=352), which is not reflective of true figures. The reason behind this under-diagnosis is a very significant incidence of iron deficiency anemia in study cohort, which limits the reliability of HPLC results. Reported studies from Pakistan stated that β-Thal is the most common hemoglobinopathy in our region particularly β-TT.^9^ In contrast to our results, Waheed et al. reported the overall frequency of hemoglobinopathies 28.4% which is exactly twice of the frequency observed in our study.^10^ Another report from Khyber Pakhtunkhwa (KPK) showed 48.9% of the study subjects suffering from various hemoglobinopathies.^11^ The underlying reason for the relatively low frequency observed herein might be the misdiagnosis of β-TT as iron deficiency anemia (IDA). Since both diseases involve overlapping morphological features i.e. hypochromia and microcytosis.^12^ Though our study based on a very large number of cases but relatively lower frequency of hemoglobinopathy reported herein may be secondary to sample bias. Majority (68%) of the studied cases were females, visited in antenatal clinics. Iron deficiency is more prevalent in the females of reproductive age and can limit the diagnostic accuracy of β-TT and HbE trait by conventional testing methods. Iron profile of study cohort clearly indicates that high frequency of iron deficiency anemia adversely affected the overall results and led to under-diagnosis in some heterozygous cases.

In contrast to our study, reported literature show equal distribution of males and females in carriers of hemoglobinopathy while some studies showed male predominance.^13^,^14^ Again, this difference in gender distribution could be due to the majority of participants from antenatal clinics. As we know, the age at diagnosis depends upon the disease severity; homozygous patients particularly β-TM usually present and diagnose earlier while heterozygous states like β-TT, because of asymptomatic nature are identified incidentally or by family screening studies.

Similar to other studies published from our region, β-TT (155/352, 44%) was the most common hemoglobinopathy followed by β-TM (114/352, 32.4%) in this study. Though, β-TT occurs widely including Middle East, Central Africa, Southwest Europe, and the Mediterranean region, half of the burden worldwide is carried by Southeast Asian countries.^15^,^16^ Another study reported contrasting results with sickle cell trait being a major hemoglobin disorder identified in their region.^17^ The variations in the results are endorsed by the past records which indicated the variable geographical distribution of hemoglobinopathies.^18^ Among variant hemoglobinopathies, sickle-β Thalassemia is most common while compound heterozygosity for HbS and HbD is observed as the least common entity. However, in our region β-Thal and HbE disease are more frequent. While sickle cell disorders are most common in African region, Saudi Arabia and in some tribal populations of central India. Prevalence of Hb S gene is up to 25% in eastern Saudi Arabia however it is comparatively low in our population.^17^

Hemoglobin E is a beta chain variant which is common in South East Asia with the highest frequency in Thailand i.e. 14-15%. Similar to beta thalassemia trait, presence of Hb E is masked by IDA.^18^ In contrast to other local studies, HbE disease frequency is relatively higher in our study. It could be due to the diagnostic challenge to distinguish HbE from HbA2 as both co-elute at the same retention time. Considerable skills and experience are needed to interpret such cases. Hb D is also a beta chain variant, its highest incidence is reported amongst Sikhs in the Punjab, Indian region. It is important to distinguish Hb D Punjab from other alpha and beta chain variants with similar electrophoretic mobility or elution time. The most common alpha chain variant with the potential to be confused with HbD is HbG Philadelphia which is exceptionally rare in our geographical region.^19^ In summary, variant hemoglobinopathies, whether in homozygous or heterozygous state, are found to be relatively less prevalent in our cohort.

As stated above, diagnosis of hemoglobinopathies rests on combination of clinical history, CBC parameters, HPLC results as well as biochemical profile particularly in our scenario where incidence of nutritional deficiency is very high. HbS disorders usually have normal red cell indices while all beta chain variants present with low MCV, MCH, MCHC and raised RDW therefore simultaneous investigation of iron profile is very helpful. Although in individuals who do not have β-TT, megaloblastic anemia resulting either from folic acid deficiency or vitamin B12 deficiency causes a significant rise in HbA2 percentage.^20^ Due to lack of awareness regarding the impact of biochemical profile on HPLC results, it is not a common practice to investigate iron, vitamin B12 and folate levels while suspecting hemoglobinopathy. It is also evident by our study where biochemical profile was investigated only in a limited number of patients. The diagnosis of β-TT in patients with concurrent iron deficiency may be confounded by reduced HbA_2_. Hence, iron deficiency should be identified and treated in patients with strong suspicion of β-TT.^21^-^23^

There are some other inherited and acquired causes of increased or decreased concentration of HbA2 which should be considered before making a diagnosis. DNA analysis and family studies are vital tools in such cases. Though molecular characterization is the most reliable way to address the issue of under-diagnosis but it is not widely available therefore cannot be considered as first line investigation in countries like Pakistan where expertise as well as cost is a major challenge.

### Limitations:

The results of this study are biased as only selected samples were tested using conventional methods. Additionally, major proportion of study cohort is comprised of samples received from antenatal clinics. Moreover, high incidence of iron deficiency anemia in the population as well as in the study cohort is another limiting factor which could be a reason of under diagnosis.

## CONCLUSION

This study was conducted to investigate the burden of hemoglobin disorder in our population. Since the overall prevalence is lower than the predicted rate hence highlights the aspects of under/misdiagnosis. The β-TT was observed to be the most prevailing hemoglobinopathy followed by β-TM. The SCD, sickle-β Thal, HbE and HbD etc. were among the hemoglobin variants identified in studied cohort. Hemoglobinopathies can be prevented by population screening, genetic counseling, and prenatal diagnosis. We hope that the present study will be helpful in raising the awareness regarding the burden of the hemoglobinopathies and in turns pave the way for the design of future policies in regards to screening practices and diagnostic measures.
